# An immunocompetent migrant presenting with neurosyphilis with an unusual unilateral papillitis: a case report

**DOI:** 10.1186/2047-783X-17-3

**Published:** 2012-02-14

**Authors:** Paolo Turchetti, Fernanda Pacella, Elena Pacella, Concetta Mirisola, Ilaria Uccella

**Affiliations:** 1 National Institute for Health, Migration and Poverty (INMP/NIHMP), Rome, Italy; 2 Ophthalmological Science Department, University 'La Sapienza', Rome, Italy

**Keywords:** migrant, papillitis, syphilis, treponema

## Abstract

Unilateral papillitis caused by *Treponema pallidum *was found in an immunocompetent homosexual patient with severe vision loss who had received previous antibiotics treatment. Syphilis-related ocular manifestation is more common in the early stages of the disease and it can be associated with a central nervous system localization. In this patient, neurosyphilis was diagnosed on the basis of clinical and laboratory findings. Optical examination revealed unilateral papillitis in the left eye and no relative afferent pupillary defects. The patient underwent visual field examinations with conventional perimetry using the 30-2 program of the Humphrey Visual Field Analyzer, which indicated a blind spot enlargement in the left eye. Optical coherence tomography, visual evoked potentials (VEP), and fluorescein angiograms revealed inflammation of the optic nerve head with edematous and blurred margins. A reactive *T. pallidum *hemagglutination assay with low rapid plasma reagin (RPR) serum titer was performed; an HIV antibody test and MRI of the orbits and head with contrast gave negative results. Resolution of the ocular inflammation after intravenous penicillin treatment was obtained. The reported case illustrates the importance of early recognition of this treatable disease. The rise of syphilis, especially in urban areas, necessitates a high level of suspicion when dealing with patients with intraocular inflammation of unknown origin. Lues serology should be incorporated into routine laboratory diagnostics to aid in the detection of such cases. Considering the re-emergence of syphilis, screening of migrants from countries with high syphilis seroprevalences should be recommended.

## Introduction

Syphilis represents a global health issue, with an estimated 12 million people infected every year; this infectious disease is prevalent in Africa, Asia and South America. In European countries, syphilis is considered a re-emerging infectious pathology. The European Centre for Disease Prevention and Control (ECDC) has reported that the overall number of syphilis cases increased substantially in most Western European countries between 1998 and 2007, mostly among men [1]_._

In recent years, most of the reported syphilis cases have been associated with HIV infection [2-5].

In 2007, Italy registered 720 cases of syphilis, 561 among males. No data exist about migrants. The complexity of syphilis diagnosis based on clinical and serological findings is already subject of scientific discussion, and is leading to the revision of treatment guidelines [6,7].

Severe vision loss due to *Treponema pallidum *causing syphilis is a rare manifestation of this disease in the antibiotic era, especially in developed countries [8,9]. The most common ocular manifestation of this infection is uveitis, occurring in 2.5 to 5% of patients with tertiary syphilis [10]. Other ocular manifestations are focal retinitis, papillitis, iritis, keratic precipitates, periphlebitis, vitritis, and serous and exudative retinal detachment [11,12].

The ability of syphilis to mimic different ocular disorders can lead to misdiagnosis and delay in the administration of appropriate antimicrobial therapy, particularly in persons who receive inadequate penicillin therapy. To the best of our knowledge, few cases of unusual neurosyphilis presentation have been reported in the literature. In the present work, a case of ocular and atypical neurosyphilis in an HIV-negative patient is described.

## Case report

A 38-year-old Brazilian transgender patient attending the outpatient department of the National Institute for Health, Migration and Poverty (NIHMP), presented with a sudden decrease of visual acuity in the left eye (visual best-corrected acuity (VBCA) 20/70). The patient's history revealed a previous treatment with antibiotics for a dental infection. The last event of unprotected sexual intercourse dated back to 1 year previously.

**Figure 1:**
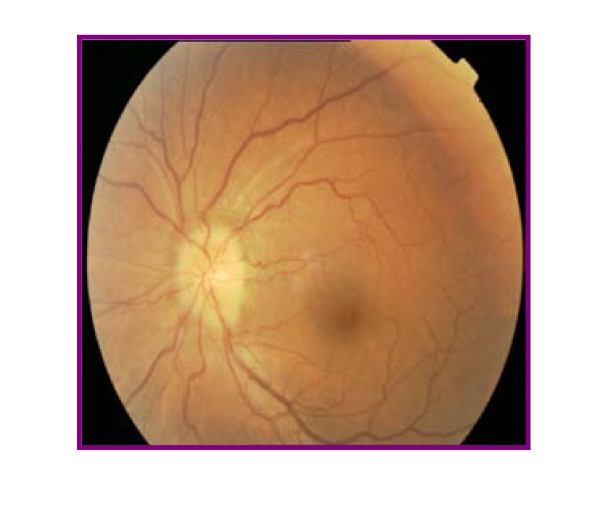
**Fundus appearance of the left eye at initial presentation**.

**Figure 2:**
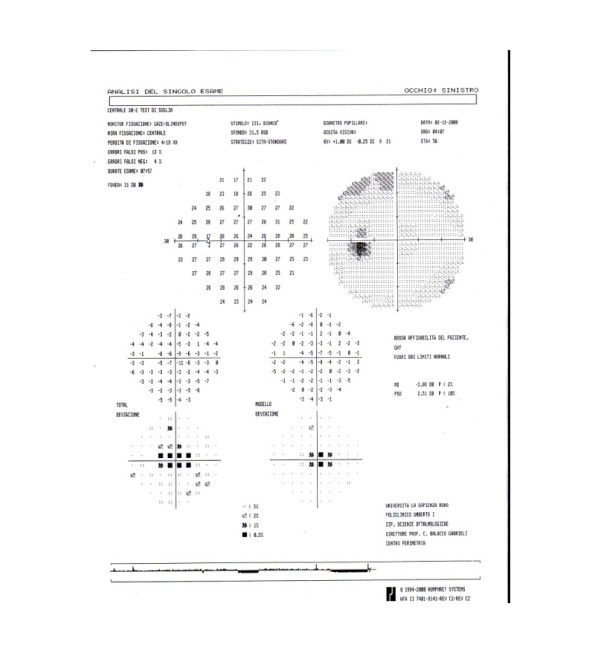
**The 30-2 program of the Humphrey Visual Field Analyzer that indicates a slight central sensitivity loss or a paracentral scotoma in the left eye (see also Figure 3)**.

**Figure 3:**
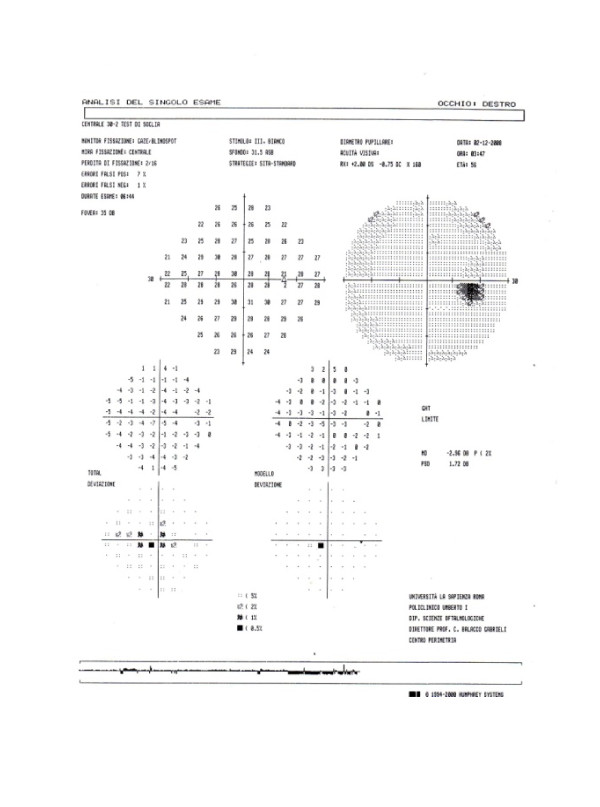
**The 30-2 program of the Humphrey Visual Field Analyzer that indicates a slight central sensitivity loss or a paracentral scotoma in the left eye (see also Figure 2)**.

An optical examination revealed unilateral papillitis in left eye (Figure 1) and no relative afferent pupillary defect (RAPD). The patient underwent visual field examinations with conventional perimetry using the 30-2 program of the Humphrey Visual Field Analyzer that indicated a slight central sensitivity loss or a paracentral scotoma in the left eye (Figures 2 and 3).

**Figure 4:**
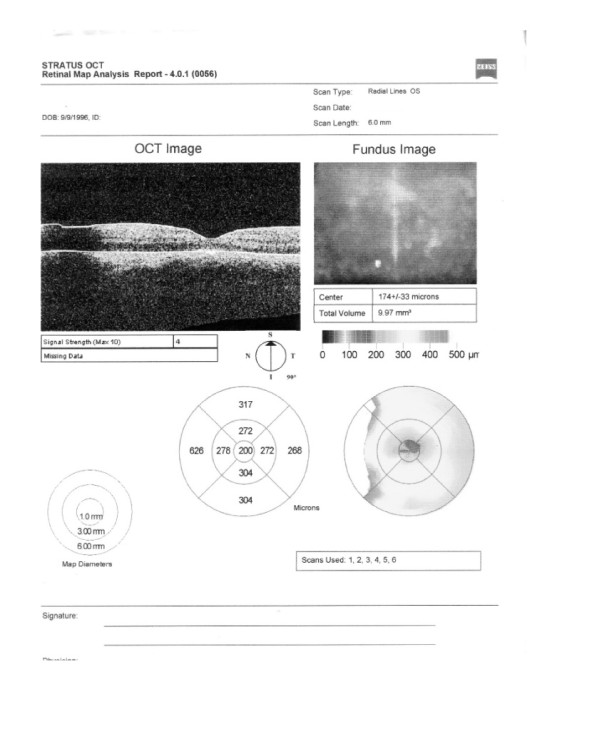
**Optical coherence tomography (OCT) scan of the left eye at initial presentation**. There was some thickening of the retinal pigment epithelium (RPE) layer with the inflammation of the optic nerve head with edematous and blurred margins.

**Figure 5:**
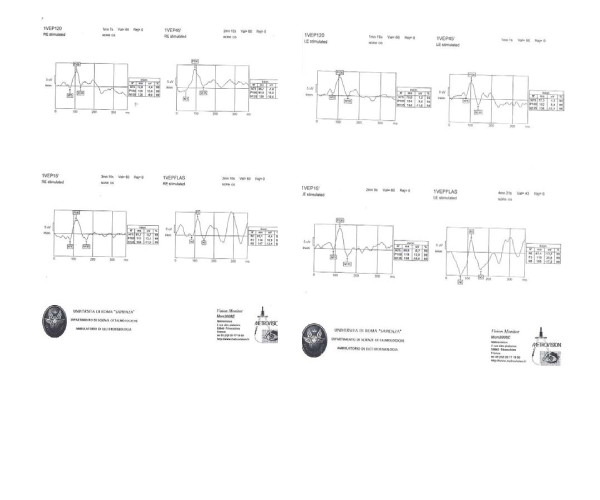
**The visual evoked potentials (VEP) revealed a decrease in VEP amplitude with normal latency in the left eye**.

**Figure 6:**
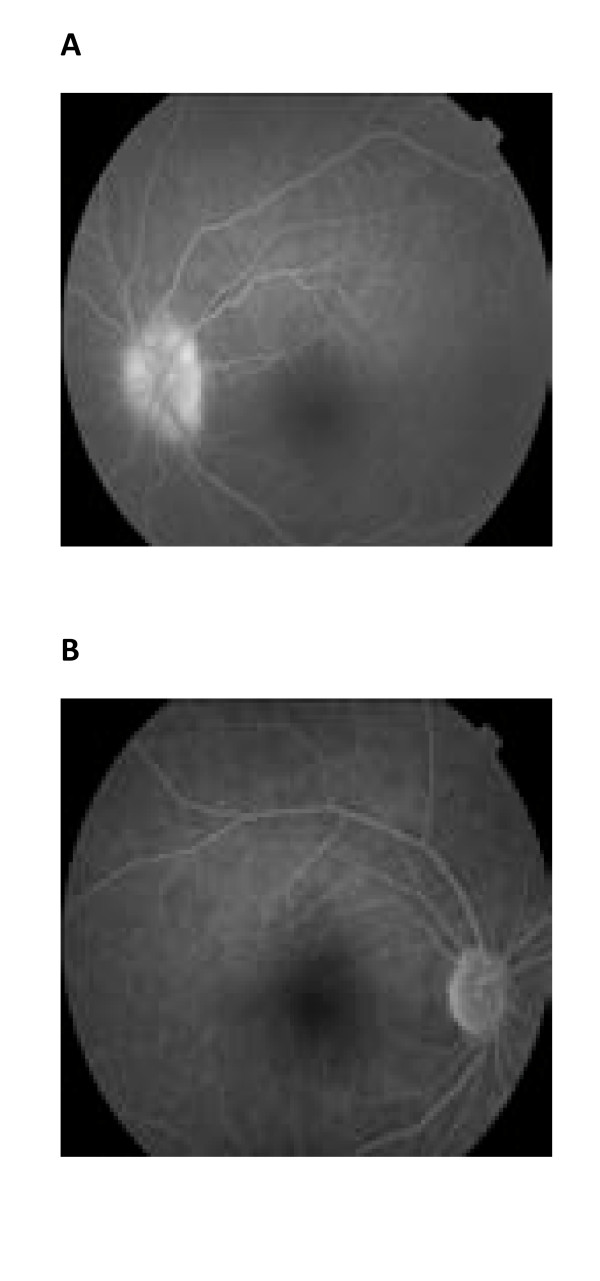
**(A) Fundus fluorescein angiogram at presentation of syphilitic papillitis**. **(B) **Fundus fluorescein angiogram 4 weeks after treatment.

An optical coherence tomography (OCT) scan of the left eye showed some thickening of the retinal pigment epithelium (RPE) layer at initial presentation (Figure 4). The visual evoked potentials (VEPs) revealed a decrease in VEP amplitude with normal latency (Figure 5). Fluorescein angiograms (FA) (Figure 6A, B) revealed inflammation of the optic nerve head with edematous and blurred margins.

On clinical examination, neurological symptoms were excluded. A reactive *Treponema pallidum *hemagglutination assay (TPHA) with low rapid plasma reagin (RPR) titer was performed. An HIV antibody test gave negative results. Different internal localization of syphilis (gumma) and mucocutaneous lesions were absent. Blood tests to eliminate the suspicion of other infections and systemic disorders were performed. An MRI of the orbits and head with contrast did not show signal alteration.

The patient was treated with 12 million units of aqueous penicillin G intravenously daily (3 million units intravenously every 4 h) for 14 days with resolution of the anterior segment and the optical nerve inflammation. As the antibiotic therapy was administered, the patient's visual acuity gradually fully recovered. He was unfortunately lost to follow up because our hospital accepts and treats patients who have no residence permit and who often are afraid to report for checks.

## Conclusions

Papillitis, also known as optic neuritis, is characterized by inflammation and deterioration of the portion of the optic nerve known as the optic disc. This localization is a relatively rare ocular manifestation of syphilis and its incidence is high in HIV-positive individuals [13-15].

Uveitis is more common in early syphilis [16] and may be accompanied by neurosyphilis. Among patients with secondary syphilis, about 5% present with chorioretinitis and 50% of these with bilateral lesions [16,17].

Moreover, granulomatosus anterior uveitis could be related to other infectious diseases and autoimmune disorders (tuberculosis, herpes viruses, Lyme disease, Behçet's disease, sarcoidosis) that have to be excluded.

In our patient's case, the diagnosis of papillitis due to *T. pallidum *was performed on the basis of serological positive test and clinical ocular manifestations. The consideration that our patient was young and a congenital infection, which can be asymptomatic in 50% of cases and is very rare, could not be excluded (30% to 40% of untreated persons) [18,19]. Other stages than tertiary syphilis were excluded because genital and mucocutaneous lesions were absent. The debate about performing lumbar puncture on HIV-negative patients with syphilis is still open [20,21]. The absence of HIV positivity and of neuroradiological alterations and the previous antibiotic treatment allowed us to avoid lumbar puncture. The treatment of papillitis was managed as neurosyphilis [22,23]. A report on posterior ocular manifestation of syphilis with spontaneous resolution was published recently [24]. Furthermore, experts do not recommend the treatment for neurosyphilis in HIV-negative individuals in whom *T. Pallidum *is found on CSF without other abnormalities [20]. On the basis of the reported case, we theorized that previous antibiotic therapy for other diseases in patients with syphilis may alter the natural history, the course, and the clinical manifestations of the pathology. In the available medical literature, only a few cases of papillitis due to *T. pallidum *are described in immunocompetent persons [25-28]. No report exists of unilateral papillitis as the first and only manifestation of syphilis in HIV-uninfected migrants. Despite the existence of programs aimed at controlling sexually transmitted diseases in Europe, syphilis screening among migrants is infrequently recommended. In Europe, studies on the seroprevalence of syphilis in migrants are generally conducted on migrant sex workers [29,30]. In Spain, sex workers from South America with syphilis antibodies were reported in 3% of cases [31]. In 2009, an increase in the incidence of ocular syphilis in homosexual HIV-positive individuals was observed in Italy [32]. In Portugal, where African migrants are prevalent, syphilis is reported in 4.1% of sexually transmitted infections [33].

Syphilitic ocular manifestation should be considered in all patients with or without concurrent HIV infection or in any case of vision loss of uncertain origin with unexplained ocular inflammation. Syphilis screening through ocular examination should be performed to aid in the detection of such cases (among migrants from countries registering high prevalence of this sexually transmitted disease).

## Consent

Written informed consent was obtained from the patient for publication of this case report and any accompanying images. A copy of the written consent is available for review by the Editor-in-Chief of this journal.

## Competing interests

The authors declare that they have no competing interests.

## Authors' contributions

PT conceived the case report and participated in the drafting of the manuscript; FP performed the ophthalmic emergency visit and clinic follow up; EP performed the OCT; CM participated in the drafting of the manuscript; IU participated in its design and coordination and drafting of the manuscript. All authors read and approved the final manuscript.
